# Evaluation of the Cervical Physiotherapeutic Treatment Needs, Work Ergonomics, and Necessity for Physical Activity Among Students of Dentistry at a Medical University. A Pilot Study

**DOI:** 10.3389/fpsyg.2020.559657

**Published:** 2020-10-22

**Authors:** Joanna Kuć, Małgorzata Żendzian-Piotrowska

**Affiliations:** ^1^Department of Prosthodontics, Medical University of Bialystok, Bialystok, Poland; ^2^Department of Hygiene, Epidemiology and Ergonomics, Medical University of Bialystok, Bialystok, Poland

**Keywords:** burnout, dentistry, ergonomics, health needs, physical activity, stress, well-being

## Abstract

**Introduction:**

A lot of general dental practitioners suffer from job-related health problems. They are more prone to numerous different physical and psycho-emotional triggers that aggravate their working conditions.

**The Aim:**

The aim of the study was to evaluate cervical physiotherapeutic treatment needs, daily ergonomic habits and the necessity for physical activity among students of dentistry at a medical university.

**Materials and Methods:**

112 generally healthy dentistry students (73 women, 39 men) were enrolled for the study. The age range was 20–32, with an average of 22.88 ± 2.7. The study included subjects in their second to fifth year of studies. The survey was conducted with respect to the questionnaires about possible physiotherapeutic treatments, physical activities, imaging tests and diagnoses as well as statements regarding the respondents’ knowledge and ergonomics habits. To estimate the association between the risk factor and neck disability as well as chronic pain, the Neck Disability Index, Perceived Stress Scale and the Graded Chronic Pain Scale were additionally applied.

**Results:**

35.71% of dentistry students had undergo physiotherapy in the preceding year. 26.79% of the respondents declared that they underwent rehabilitation on a regular basis. The most common reasons for treatment were complaints of the cervical (7.14%), lumbar (3.57%), and thoracic spine (1.79%) as well as a combination of these symptoms. 6.25% of the subjects had started rehabilitation due to muscle relaxation. 34.82% of the study participants did not do any physical activity. Females exercised more often than males (*p* = 0.02; 1–β = 0.65). Therapeutic exercises contributed to the improvement of well-being of 56% of students. 64.29% of the respondents chose a sitting position during pre-clinical and clinical classes and 26.79% of the subjects worked in sitting and standing postures.

**Conclusion:**

26.79% of dentistry students required periodic therapeutic rehabilitation. 34.82% of the respondents were advised to implement systematic physical activity. Due to professional conditions, dental students are exposed to an early cervical spine disorders. The main factor influencing an early onset of such dysfunctions is increased stress level. An average level of pro-health awareness may be the reason for elevated cost of rehabilitation among this group of patients.

## Introduction

Occupational hazard is associated with the risk of job-related health problems. It can refer to the working environment, materials, substances, processes or situations that initiate or promote workplace incidents and/or disabilities. The role of occupation in the dynamics of health and sickness was first described by Bernardo Ramazzini in the 18th century (Fasunloro and Owotade, 2004).

A lot of general dental practitioners suffer from job-related health problems (Myers and Myers, 2004). They are more prone to numerous different physical and psycho-emotional triggers that aggravate their working conditions (Myers and Myers, 2004; Moodley et al., 2018). A static position, typically adapted during dental procedures, result in a 10% higher energy consumption, increased heart rate, overload on the cardiovascular and osteoarticular systems, overcharging of the body with a functionally-tailored reduced support surface, pain and discomfort in the calves (Anghel et al., 2007). The evaluation of momentary workloads does not reveal the amount of the experienced cumulative trauma (Newell and Kumar, 2005). In the long term, tissue load becomes a health-modulating factor (Newell and Kumar, 2005). Work-related static posture leads to the development of musculoskeletal disorders. Over 70% of dental students reported pain around the third year of their studies (Rising et al., 2005). 87.2% of the dentists manifested at least one symptom of musculoskeletal disorder in the last year of their clinical practice (Leggat and Smith, 2006). The factors promoting such problems include: constantly repeated movements, twisting of the trunk, multiple daily cervical flexion as well as strong arm movement (e.g., working with arms above the shoulder) and sedentary behavior (e.g., long time in a seated position without breaks; always working in the same position) (Valachi and Valachi, 2003a,b; Presoto and Garcia, 2016; Kuć and Żendzian-Piotrowska, 2020).

The pain within the musculoskeletal system appears at the time when dentists begin professional studies and remain at the university for the duration of their practice (Diaz-Caballero et al., 2010). A typical clinical pattern includes neck, shoulder, arm and wrist pain (Moodley et al., 2018). Complaints about the lumbar spine are also typical (Moodley et al., 2018). Functional changes are often manifested by a posture with the head inclined forward, loss of cervical lordosis and shoulder girdle protraction (Kuć and Żendzian-Piotrowska, 2020). Due to the common interface of the cervical spine and the trigeminocervical nucleus, many neurophysiological consequences within the orofacial area can be expected (Bogduk, 2001; Olivo et al., 2010).

It is known that muscle tension increases even despite maintaining the right posture and ensuring optimal ergonomics, which results in ischemia and hypomobility of the joints. This is related to long working hours (Valachi and Valachi, 2003a,b). The consequences of limited range of cervical spine motion (e.g., limited flexion, extension, rotation), muscle imbalance (size or strength discrepancy between right and left muscle groups), proprioception impairment (dysfunction of feedback between sensory receptors and the nervous system), seemingly idiopathic fatigue and pain (e.g., neck pain, headache, multi-site musculoskeletal pain), cause an urgent need for physical activity in relation to ergonomics (Letafatkar et al., 2019). The main goal is to prevent progression of the said changes to chronic phase of disability (Letafatkar et al., 2019).

Another important job-related health hazard of dentists concerns their psychological condition. In many cases, an occupational burnout syndrome occurs as a consequence of chronic emotional and interpersonal stressors (Maslach and Jackson, 1981). Burnout is defined as a negative work-related psychological condition associated with physical fatigue, emotional exhaustion and lack of motivation (Freudenberger, 1974). It appears as a significant risk factor of depression (Hakanen and Schaufeli, 2012). It can also adversely affect physical health, e.g., by causing cardiovascular and musculoskeletal diseases (Honkonen et al., 2006). There is a direct link between burnout of the dental health professionals, their low personal efficiency and a tendency to put patients at greater risk (Panagioti et al., 2018). Typical triggers of occupational stress among dentists include time pressure, work overload, absorbing, difficult and/or delayed patients, problems with staff, equipment or dental materials, as well as daily routine (Chohan et al., 2020). To prevent signs of burnout, attention should be paid to physical activity and work ergonomics should be provided (Gorter et al., 2000). It is also well known that the avoidance of emotional, mental and behavioral mental disabilities is an interdisciplinary subject that requires the implementation of numerous different strategies (O’Connell et al., 2009).

## The Aim

The aim of the study was to evaluate cervical physiotherapeutic treatment needs, daily ergonomic habits and the necessity for physical activity among students of dentistry at a medical university.

## Materials and Methods

### The Subjects and Sample Size

One hundred and twelve generally healthy students (73 women and 39 men) of the faculty of medicine, division of dentistry of the Medical University of Bialystok, Poland were enrolled for the study. The age range was 20–32, with an average of 22.88 ± 2.7 (Mean_female group_ = 22.99 ± 2.65; Mean_male group_ = 22.67 ± 2.80). The study included subjects in their second to fifth year of studies. The research was conducted in the period from January to April 2018.

### General Description of the Method

The survey was conducted with the use of the following:

• A questionnaire about possible physiotherapeutic treatments and physical activities, including the following questions:
  Which forms of physical therapy did you use within the last year? How often do you use rehabilitation procedures? What was the reason/cause for undertaking rehabilitation? How often do you use therapeutic exercises (e.g., yoga, stretching, isometric exercises, etc.)? How do the therapeutic exercises you use affect you?
• A questionnaire about possible imaging tests, diagnosis and confirmation of dysfunctions by a doctor or physiotherapist, including the following questions:
  Which types of diagnostic imaging tests (Magnetic Resonance Imaging—MRI, Computed Tomography—CT, blood flow volume of carotid and vertebral arteries) were performed in your case and in which section of the spine? Which of the diagnoses were confirmed by a doctor or physiotherapist?
• Statements of the respondents regarding their knowledge and habits in the field of ergonomics, including the following questions:
  What is the right definition of head protraction? Which body position do you assume most frequently when you use a laptop, Tablet or read a book? Which body position is the most common for you during your clinical practice at the university? What is your most common body position when you treat tooth decay located on the palatal side of the upper incisors?
• The Neck Disability Index (NDI), Perceived Stress Scale (PSS-10) and Graded Chronic Pain Scale (GCPS v. 2.0) were additionally applied to estimate the association between the risk factor and neck disability as well as chronic pain.

The response rate to the questionnaires amounted to 100%. The presented subjects and sample size remain in accordance with the study group described in the previous publication (Kuć and Żendzian-Piotrowska, 2020).

### Statistical Analysis

A statistical analysis was performed using Statistica 13.1 (Statsoft Inc. United States) and G Power v. 3.1.9.4 software. The measure of central tendency (mean) and variation (standard deviation) were used to describe the distribution of quantitative variables (age of the respondents).

The multivariate frequency distribution of the variables was demonstrated in 2 × 2 contingency tables. The percentage within columns (percentage of the entire group, the female group and the male group, respectively) was also presented.

Due to the relatively small sized sample (expected number of frequencies ≤5), Fisher’s exact one-tailed test was applied to compare differences in frequency with respect to the gender. Differences with *p* < 0.05 were considered statistically significant.

*Post hoc* power analysis was performed using G Power v.3.1.9.4 software. Power (1-β) was assessed as the function of the population effect size, the number of cases (*n*) and α. The levels of power were presented with respect to the comparison of the differences between females and males in dichotomous approach of the examined aspect (e.g., using vs. not using physiotherapy; reasons vs not applicable; using vs not using therapeutic exercises; improvement of mood and physical condition vs all others).

Multiple linear regression models for NDI (Neck Disability Index) and GCPS v. 2.0 (Graded Chronic Pain Scale) estimation were developed by selecting the variables that contributed significantly to the evaluation of NDI and GCPS.

### Ethical Approval

The study was performed after obtaining the consent of the Bioethics Committee of the Medical University of Bialystok, Poland no. R-I-002/513/2017. Participation in the study was voluntary and anonymous. Respondents received exhaustive information about the purpose of the research and had the right to terminate their participation at any time with no repercussions.

## Results

One hundred and twelve people participated in the study, including 73 women and 39 men. The research group included 25% of all dentistry students. 33 (29.46%) of the respondents had been given massages in the preceding year. 13 (11.61%) students had undergone manual therapy. Four people (3.57%) had benefited from physical therapy and kinesiotaping. 72 (64.29%) subjects had not participated in any of the forms of physiotherapy (Table 1). There were no statistically significant differences in the frequency of having all kinds of physiotherapy in the preceding year with respect to gender (*p* > 0.05, 1-β < 0.4) (Table 1).

**TABLE 1:** Type of physiotherapy undertaken within the last year; frequency and reason for using rehabilitation procedures in the entire group (*n* = 112), the female group (*n* = 73), and the male group (*n* = 39).

				Comparison with respect to gender	
	
	Entire group	Female group	Male group	Fisher’s exact unilateral test	
	
	*n* = 112	*n* = 73	*n* = 39	*p*-value	1-β
**Type of physiotherapy undertaken in the last year**					
Manual therapy	13 (11.61%)	7 (9.59%)	6 (15.38%)	0.27	0.16
Massage	33 (29.46%)	25 (34.25%)	8 (20.51%)	0.10	0.37
Physical therapy	4 (3.57%)	3 (4.11%)	1 (2.56%)	0.57	0.01
Kinesiotaping	4 (3.57%)	3 (4.11%)	1 (2.56%)	0.57	0.01
Others	2 (1.79%)	1 (1.37%)	1 (2.56%)	–	–
Lack of use of physiotherapy	72 (64.29%)	45(61.64%)	27 (69.23%)	0.28	0.15

**Frequency of using rehabilitation procedures**				**Using vs. not using physiotherapy**	

Once a week	3 (2.68%)	3 (4.11%)	0 (0.00%)	0.28	0.11
2–3 times a month	2 (1.79%)	1 (1.37%)	1(2.56%)		
1 series of treatments within 6 months	5 (4.46%)	3 (4.11%)	2 (5.13%)		
1 series of treatments within 12 months	11 (9.82%)	8 (10.96%)	3 (7.69%)		
1 series of treatments within 24 months	9 (8.04%)	6 (8.22%)	3 (7.69%)		
Lack of use of physiotherapy	82 (73.21%)	52(71.23%)	30 (76.92%)		

**The reason for undertaking rehabilitation**				**Reasons vs. not applicable**	

Cervical spine pain	8 (7.14%)	4 (5.48%)	4(10.26%)	0.39	0.09
Lumbar spine pain	4 (3.57%)	4 (5.48%)	0 (0.00%)		
Thoracic spine pain	2 (1.79%)	1 (1.37%)	1 (2.56%)		
Headaches	1 (0.89%)	1 (1.37%)	0 (0.00%)		
Injuries	0 (0.00%)	0 (0.00%)	0 (0.00%)		
Cervical and lumbar spine pain	3 (2.68%)	3 (4.11%)	0 (0.00%)		
Cervical, lumbar and thoracic spine pain	1 (0.89%)	1 (1.37%)	0 (0.00%)		
Cervical spine pain and headache	1 (0.89%)	1 (1.37%)	0 (0.00%)		
Lumbar and thoracic spine pain	2 (1.79%)	2 (2.74%)	0 (0.00%)		
Lumbar spine pain and injuries	1 (0.89%)	1 (1.37%)	0 (0.00%)		
Cervical spine pain and shoulder pain	1 (0.89%)	1 (1.37%)	0 (0.00%)		
Cervical spine pain, thoracic spine pain, shoulder pain and calf pain	1 (0.89%)	0 (0.00%)	1 (2.56%)		
Muscle relaxation	7(6.25%)	3 (4.11%)	4 (10.26%)		
Not applicable	80 (71.43%)	51 (69.86%)	29 (74.36%)		

*% percentage within column (% entire group; % female group; % male group, respectively).*

82 (73.21%) respondents declared that they did not attend any rehabilitation at all (Table 1); 11 students (9.82%) undertook therapy once a year; 9 (8.04%) people had had 1 series of physiotherapeutic treatments within the preceding 24 months; 3 (2.68%) participants benefited from physiotherapy once a week; 2 (1.79%) people underwent rehabilitation 2–3 times a month (Table 1). There were no statistically significant differences in the frequency of physiotherapeutic procedures with respect to gender (*p* = 0.28, 1-β = 0.11) (Table 1).

The most common reason for attending rehabilitation among the entire study group (*n* = 112) were complaints about the cervical (7.14%), lumbar (3.57%), thoracic spine (1.79%) as well as a combination of these (8.92%) (Table 1). Seven people (6.25%) had started rehabilitation for the purpose of relaxation (Table 1). No statistically significant differences were noted between males and females (*p* = 0.39, 1-β = 0.09) (Table 1).

39 (34.82%) respondents did not do any physical activity (Table 2). Nobody exercised every morning and evening. 14 (12.5%) students trained regularly every few days. 29 (25.89%) subjects were physically active unsystematically, and other 24 (21.43%) worked out very rarely (Table 2). Physical activity was more often performed by women than men (*p* = 0.02; 1-β = 0.65) (Table 2). In the case of 62 (56%) students, therapeutic exercises contributed to the improvement of their well-being (Table 2). 1 (0.89%) person reported that physical activity adversely affected their health. For 2 (1.79%) subjects, therapeutic exercises caused cervical spine pain. There were no statistically significant differences in mood and fitness improvement with respect to gender (*p* = 0.20; 1-β = 0.36) (Table 2).

**TABLE 2:** The frequency of using therapeutic exercises (e.g., yoga, stretching, isometric exercises, etc.) and the influence of commonly used therapeutic exercises in the entire group (*n* = 112), the female group (*n* = 73), and the male group (*n* = 39).

				Comparison with respect to gender	
	
	Entire group	Female group	Male group	Fisher’s exact unilateral test	
	
	*n* = 112	*n* = 73	*n* = 39	*p*-value	1-β

The frequency of using therapeutic exercises				Using vs. not using therapeutic exercises	
Every morning and evening	0 (0.00%)	0 (0.00%)	0 (0.00%)	0.02*	0.65
Every morning	1 (0.89%)	0 (0.00%)	1 (2.56%)		
Every evening	5 (4.46%)	4 (5.48%)	1 (2.56%)		
Systematically every few days	14 (12.50%)	10(13.70%)	4 (10.26%)		
Irregularly	29 (25.89%)	20 (27.40%)	9 (23.08%)		
Very rarely	24 (21.43%)	19 (26.03%)	5 (12.82%)		
Lack of using therapeutic exercises	39 (34.82%)	20 (27.40%)	19 (48.72%)		

**Influence of commonly used therapeutic exercises**				**Improvement of mood and physical condition vs. all others**	

Improvement of mood	30 (27.00%)	21 (29.00%)	9 (23.00%)	0.20	0.36
Improvement of physical condition	12 (11.00%)	6 (8.00%)	6 (15.00%)		
Improvement of mood and physical condition	20 (18%)	16 (22.00%)	4 (10.26%)		
Deterioration of condition	1 (0.89%)	1 (1.37%)	0 (0.00%)		
Cause of cervical spine pain	2 (1.79%)	1 (1.37%)	1 (2.56%)		
No effect on the body	3 (2.68%)	3 (4.11%)	0 (0.00%)		
Not applicable	44 (39.29%)	25 (34.25%)	19 (48.72%)		

*p < 0.05 statistical significance. % percentage within column (% entire group; % female group; % male group, respectively).

The students reported having 22 high-imaging tests performed (Table 3), 6 (5.35%) computed tomography (CT) scans and 2 (1.79%) magnetic resonance imaging (MRI) tests of the head. 4 (3.57%) MRI tests and 1 CT scan (0.89%) of the cervical spine were performed. 5 (4.46%) students declared imaging of the lumbar spine, while other 3 (2.68%) reported imaging of the thoracic region (Table 3). A statistically significant higher prevalence of high-imaging tests was noted among females compared to males (*p* = 0.02; 1-β = 0.50) (Table 3).

**TABLE 3:** The frequency of diagnostic imaging tests performed by the students in the entire group (*n* = 112), the female group (*n* = 73), and the male group (*n* = 39).

				Comparison with respect to gender diagnostic imaging test vs. lack of diagnostic imaging test	
	
	Entire group	Female group	Male group	Fisher’s exact unilateral test	
	
	*n* = 112	*n* = 73	*n* = 39	*p*-value	1-β
**Diagnostic imaging test**					
MRI of cervical spine	4 (3.57%)	4 (5.47%)	0 (0.00%)	0.02*	0.50
CT of cervical spine	1 (0.89%)	1 (1.37%)	0 (0.00%)		
MRI of the head	2 (1.79%)	2 (2.73%)	0 (0.00%)		
CT of the head	6 (5.35%)	6 (8.21%)	0 (0.00%)		
MRI or CT of lumbar spine	5 (4.46%)	4 (5.47%)	1 (2.56%)		
MRI or CT of thoracic spine	3 (2.68%)	2 (2.73%)	1 (2.56%)		
Blood flow volume of carotid and vertebral arteries	1 (0.89%)	1 (1.37%)	0 (0.00%)		
**^.^of which a combination were:**					
MRI and CT of cervical spine	1 (0.89%)	1 (1.37%)	0 (0.00%)		
MRI of cervical spine and MRI or CT of lumbar spine	2 (1.79%)	2 (2.74%)	0 (0.00%)		
MRI of the head and MRI or CT of lumbar and thoracic spine	1 (0.89%)	1 (1.37%)	0 (0.00%)		
MRI or CT of lumbar and thoracic spine	1 (0.89%)	1 (1.37%)	0 (0.00%)		
MRI of cervical spine and blood flow volume of carotid and vertebral arteries	1 (0.89%)	1 (1.37%)	0 (0.00%)		
None of the above	97 (86.6%)	60(82.19%)	37(94.87%)		

*^.^p < 0.05 statistical significance. % percentage within column (% entire group; % female group; % male group, respectively).*

Nobody was operated on due to cervical spine injury, cervical discopathy or its sequelae, cancer within the cervical spine, cervical spine instability, birth defects, or cervical spine degeneration.

In 99 (88.39%) students no cervical spine dysfunction were confirmed by a doctor or physiotherapist (Table 4). Three people (2.68%) were diagnosed with cervical discopathy and loss of cervical lordosis. Individual participants (0.89%) declared instability, hypomobility, degenerative changes, whiplash injury, dehydration of intervertebral discs and reduction of vertebral bodies (Table 4).

**TABLE 4:** Diagnosis confirmed by a doctor or physiotherapist in the entire group (*n* = 112), the female group (*n* = 73), and the male group (*n* = 39).

	Entire group *n* = 112	Female group *n* = 73	Male group *n* = 39
**Diagnosis confirmed by a doctor or physiotherapist**			
Cervical spine instability	1 (0.89%)	0 (0.00%)	1 (2.56%)
Cervical spine discopathy	3 (2.68%)	3 (4.11%)	0 (0.00%)
Hypermobility	0 (0.00%)	0 (0.00%)	0 (0.00%)
Hypomobility	1 (0.89%)	1 (1.37%)	0 (0.00%)
Degenerative changes of the cervical spine	1 (0.89%)	1 (1.37%)	0 (0.00%)
Loss of cervical lordosis	3 (2.68%)	2 (2.73%)	1 (2.56%)
Cervical spine injury	0 (0.00%)	0 (0.00%)	0 (0.00%)
Whiplash injury	1 (0.89%)	1 (1.37%)	0 (0.00%)
Congenital abnormalities of cervical spine	0 (0.00%)	0 (0.00%)	0 (0.00%)
Dehydration of intervertebral discs	1 (0.89%)	1 (1.37%)	0 (0.00%)
Reduction of vertebral bodies of the cervical spine	1 (0.89%)	1 (1.37%)	0 (0.00%)
Cervical spinal stenosis, osteophytes, syndesmophytes, cervical radiculopathy, cervical, narrowing of intervertebral foramen, shoulder pain, fibromyalgia, ankylosing spondylitis, tumors in the cervical spine, upper crossed syndrome, ‘’text neck”	0 (0.00%)	0 (0.00%)	0 (0.00%)
None of the above	99(88.39%)	64(87.67%)	35(89.74%)

*% percentage within column (% entire group; % female group; % male group, respectively).*

72 (64.29%) subjects provided a proper definition of head protraction (Table 5). 46 (41.07%) students reported the correct amount of load (23.33 kg) of the cervical spine at its 45° flexion (Table 5). 46 (41.07%) respondents suggested a load of 23.33 kg. There were no statistically significant differences with respect to gender (*p* > 0.05) (Table 5).

**TABLE 5:** Defining head protraction and probable load on the cervical spine in the 45° flexion in the entire group (*n* = 112), the female group (*n* = 73), and the male group (*n* = 39).

				Comparison with respect to gender	
	
	Entire group	Female group	Male group	Fisher’s exact unilateral test	
	
	*n* = 112	*n* = 73	*n* = 39	*p-*value	1-β

Definition of the head protraction				Right vs. wrong answer	
✓ Physiological position of the head in space	3 (2.68%)	3 (4.11%)	0 (0.00%)	0.14	0.03
✓ Therapeutic position of the head forced by a physiotherapist	8 (7.14%)	4 (5.48%)	4 (10.26%)		
✓ Forward non-physiological head posture^a^	72 (64.29%)	50 (68.49%)	22 (56.41%)		
✓ Combined movement resulting from extension, lateral slope and rotation of the cervical spine	4 (3.57%)	0 (0.00%)	4 (10.26%)		
✓ Combined movement resulting from flexion, lateral slope and cervical spine rotation	10 (8.93%)	6 (8.22%)	4 (10.26%)		
✓ no correct answer	15 (13.39%)	10 (13.70%)	5 (12.82%)		

**Probable load on the cervical spine in the 45° flexion**				**Right vs. wrong answer**	

11.22 kg	4 (3.57%)	3 (4.11%)	1 (2.56%)	0.08	0.42
14.36 kg	29 (25.89%)	19 (26.03%)	10 (25.64%)		
23.33 kg^a^	46 (41.07%)	34 (46.58%)	12 (30.77%)		
35.00 kg	22 (19.64%)	9 (12.33%)	13 (33.33%)		
17.69 kg	11 (9.82%)	8 (10.96%)	3 (7.69%)		

*^a^right answer; % percentage within column (% entire group; % female group; % male group, respectively).*

37 (33.03%) students indicated sitting at a desk in front of a computer/laptop or reading a book (Table 6). 30 (26.79%) people preferred lying on their back. 23 (20.54%) respondents used an armchair by raising and supporting their limbs with the footrest. 9 (8.04%) students remained in the lateral position, and 7 (6.25%) chose a position on the abdomen (Table 6). No statistically significant differences with respect to gender were found (*p* = 0.56; 1-β = 0.04) (Table 6).

**TABLE 6:** The most commonly used body positions when using a laptop, tablet or reading a book in the entire group (*n* = 112), the female group (*n* = 73), and male group (*n* = 39).

				Comparison with respect to gender sitting at the desk vs. all others	
	
	Entire group	Female group	Male group	Fisher’s exact unilateral test	
	
	*n* = 112	*n* = 73	*n* = 39	*p*-value	1-β
**Most commonly used body positions when using a laptop, tablet or reading a book**					
Lying on the back	30 (26.79%)	21 (28.77%)	9 (23.08%)	0.56	0.04
Lying on the front	7 (6.25%)	6 (8.22%)	1 (2.56%)		
Lying on the side	9 (8.04%)	6 (8.22%)	3 (7.69%)		
Sitting in an armchair with a footrest	23 (20.54%)	13 (17.81%)	10 (25.64%)		
Sitting at a desk	37 (33.04%)	24(32.88%)	13 (33.33%)		
Lying on the back and sitting in an armchair with the footrest	1 (0.89%)	0 (0.00%)	1 (2.56%)		
Lying on the back and sitting at a desk	1 (0.89%)	1 (1.37%)	0 (0.00%)		
Lying on the side and sitting at a desk	1 (0.89%)	1 (1.37%)	0 (0.00%)		
Sitting in an armchair with a footrest and sitting at a desk	1 (0.89%)	1 (1.37%)	0 (0.00%)		
Lying on the back, lying on the front and lying on the side	1 (0.89%)	0 (0.00%)	1 (2.56%)		
Lying on the back and lying on the side and sitting in an armchair with a footrest	1 (0.89%)	0 (0.00%)	1 (2.56%)		

*% percentage within column (% entire group; % female group; % male group, respectively).*

72 (64.29%) students chose a sitting position during pre-clinical classes as well as when working with patients (Table 7). 30 (26.79%) of the respondents worked in a mixed manner: assuming both sitting and standing postures. 10 (8.93%) people preferred to work by standing. No participants of the study reported four-handed work with assistance (Table 7). 92 (82.14%) students used a dental mirror and binocular vision when treating a palatal decay in the upper incisors (Table 7). There were no statistically significant differences with respect to gender (*p* > 0.05) (Table 7).

**TABLE 7:** The most commonly used body positions during clinical practice at a university in the entire group (*n* = 112), the female group (*n* = 73), and male group (*n* = 39).

				Comparison with respect to gender	
	
	Entire group	Female group	Male group	Fisher’s exact unilateral test	
	
	*n* = 112	*n* = 73	*n* = 39	*p*-value	1-β

Most commonly used body positions during clinical practice at a university				Standing work vs. all others	
Standing work	10 (8.93%)	6 (8.22%)	4 (10.26%)	0.48	0.07
Sitting work	72 (64.29%)	42 (57.53%)	30 (76.92%)		
Mixed method – standing and sitting work	30 (26.79%)	25 (34.25%)	5 (12.82%)		
4-hand work with assistance	0 (0.00%)	0 (0.00%)	0 (0.00%)		

**The most commonly used body positions during treatment of tooth decay located on the palatal side of the upper incisors**				**Use of a dental mirror and binocular vision vs. all others**	

Use of a dental mirror and binocular vision	92 (82.14%)	60 (82.19%)	32 (82.05%)	0.59	0.03
Use of a dental mirror and monocular vision	2 (1.79%)	1 (1.37%)	1 (2.56%)		
Preferring direct access to the tooth decay and using binocular vision	16 (14.29%)	11 (15.07%)	5 (12.82%)		
Preferring direct access to the tooth decay and using monocular vision	2 (1.79%)	1 (1.37%)	1 (2.56%)		
Not using a dental mirror	0 (0.00%)	0 (0.00%)	0 (0.00%)		

*% percentage within column (% entire group; % female group; % male group, respectively).*

The multiple linear regression model revealed that gender, GCPS and PSS-10 allow us to explain the approximately 30% differentiation of Neck Disability Index (NDI) (*R*^2^ = 0.31), and the prediction model is significantly better than the random one [*F*(3, 108) = 15.81; *p* < 0.00], where the average error in estimating the perceived stress level is S.E. = 3.11 (Table 8).

**TABLE 8:** Multiple linear regression model with NDI as the dependent variable and gender, GCPS v. 2.0, PSS-10 as independent variables.

	Regression coefficient (b)	SE	Standardized coefficient (β)	*t*-value	*p*-value
Intercept	0.201095	0.877537	–	0.229158	0.82
Gender	1.836016	0.627121	0.238400	2.927691	0.00*
GCPS v 2.0.	1.847878	0.469799	0.320731	3.933334	0.00*
PSS-10	0.144821	0.042154	0.282298	3.435506	0.00*
*R* = 0.55 *R*^2^ = 0.31 Adjusted *R*^2^ = 0.29; *F*(3, 108) = 15.81; *p* < 0.00 Standard error of the estimate: 3.11					

SE, standard error. *p < 0.05 statistical significance.

The second model of multiple linear regression showed that a standing position during dental procedures and physiotherapy undertaken enable us to explain the approximately 10% differentiation of the Graded Chronic Pain Scale (GCPS v. 2.0.) (*R*^2^ = 0.11), and the prediction model is significantly better than the random one [*F*(2, 109) = 6.42; *p* < 0.00], where the average error in estimating the GCPS v. 2.0. is S.E. = 0.61 (Table 9).

**TABLE 9:** Multiple linear regression model with GCPS as the dependent variable and standing position during dental procedures and physiotherapy undertaken as independent variables.

	Regression coefficient (b)	SE	Standardized coefficient (β)	*t* -value	*p*-value
Intercept	0.688645	0.073906	–	9.31787	0.00*
Standing position during dental procedures	−0.430403	0.202399	−0.192728	−2.12650	0.04*
Physiotherapy undertaken	0.354396	0.120452	0.266657	2.94222	0.00*
*R* = 0.32 *R*^2^ = 0.11 Adjusted *R*^2^ = 0.09; *F*(2, 109) = 6.42; *p* < 0.00* Standard error of the estimate: 0.61					

SE, standard error. *p < 0.05 statistical significance.

## Discussion

Occupational diseases affect dentists more often than general medicine practitioners or lawyers. Within this practical group, musculoskeletal complaints are reported 1.5 times more frequently than in other professions (Suciu et al., 2019). The incidence of cervical spondylosis is more pronounced among dentists even compared to agricultural workers (Suciu et al., 2019). This specialty is more burdensome than other medical staff which is determined by the limited intraoral operation area that promotes the adoption of the static posture (Suciu et al., 2019).

A specific dental working position leads to increased muscle tension and fatigue (Callaghan and McGill, 2001; Dantas and de Lima, 2015; Suciu et al., 2019). High static overload causes the compression of blood vessels and activity of the muscles, leading to inadequate oxygen supply, and consequently, pain. Depending on the type of cervical spine disorder, different kinds of physiotherapeutic intervention may be implemented. For example in chronic neck pain complicated by movement discoordination, education about ergonomics and lifestyle plays an important role (Blanpied et al., 2017). Physiotherapeutic procedures include mobilization of the cervical spine, strengthening exercises of the cervical and thoracic spine, mobilization of the scapular area, exercises improving strength, endurance and flexibility, functional training with elements of behavioral therapy, vestibular system rehabilitation and exercises improving eye, neck and head cooperation as well as neuromuscular coordination (Blanpied et al., 2017). The clinical goal of a manual therapy in this group of patients is maintenance of cervical lordosis in the range of 31–40° (McAviney et al., 2005). It should also be highlighted that work productivity of dentists who undergo a physical therapy is increased (Campo et al., 2008; Huang et al., 2014). This study results revealed that 64.29% of the respondents did not, in their own assessment, require any rehabilitation (Table 1). From a clinical point of view it seems to be just a matter of time. In the case of 26.79% of people, physiotherapy is undertaken on a regular basis, which may indicate a chronic, and at the same time recurrent nature of the emerging ailments (Table 1). Considering that the study focused on students, the frequency of physiotherapeutic treatments was relatively high. Due to professional predisposing factors, this situation requires monitoring.

The lack of physical activity among the respondents was not conducive to the prevention of musculoskeletal disorders (Table 2). A decreased level of physical activity is one of public health considerations of the twenty-first century (Blair, 2009; Verbunt et al., 2010). Mortality due to physical inactivity is estimated at 6%. Other leading factors include high blood pressure (13%), smoking tobacco (9%), increased levels of blood glucose (6%) as well as overweight and obesity (5%) (World Health Organization [WHO], 2017). The lack of physical activity increases the prevalence of cardiovascular diseases, diabetes and cancer (Blair, 2009). 21–25% of cases of diagnosed breast and colon cancer are physical inactivity-related. The same applies to 27% of diabetes and almost 30% of ischemic heart disease (World Health Organization [WHO], 2017). Insufficient physical activity is associated with 35 persistent conditions (Booth et al., 2011). “Exercise is Medicine” for 26 different chronic diseases (Pedersen and Saltin, 2015). Therapy exercises are prescribed with respect to psychiatric, metabolic, cardiovascular and pulmonary diseases as well as musculoskeletal disorders and cancer. In the group of dentists, a slightly increased tendency for lung cancer and overall cancer incidence exists (Simning and van Wijngaarden, 2007). Moreover, typically occurring symptoms are reflux, low back pain, headache, neck pain, osteoarthritis as well as rheumatoid and psoriatic arthritis (Nalliah et al., 2017).

In the presented study, 56% of the respondents admitted that their usual therapeutic exercises had a positive effect on their well-being (Table 2). Sharma and Golchha (2011) noted a statistically significant correlation between the number of physical activity sessions and reduction of ailments. These authors observed an improvement in health of 20–80%. Koneru and Tanikonda (2015) in turn, emphasized that yoga is the most effective form of therapy used in the prevention of neck, lumbar and shoulder girdle pain. Yoga helps strengthen the musculoskeletal system, improve flexibility, and release endorphins and hormones (Koneru and Tanikonda, 2015). In the case of dentists, in order to reduce the body’s static load resulting from straining the muscles of the back, neck and shoulders, as well as due to the proper mobilization of the muscles of the lower extremities and the torso, 30 min of daily activity is recommended (Koneru and Tanikonda, 2015). However, studies show that only 39% of dentists do sporting activities on a regular basis (Hashim and Al-Ali, 2013). It means that primary prevention among dentistry students is still required. The same applies to health promotion and a sufficient level of physical activity (Booth et al., 2011).

According to the WHO suggestions, “adults aged 18–64 should do at least 150 min of moderate-intensity aerobic physical activity throughout the week or do at least 75 min of vigorous-intensity aerobic physical activity throughout the week, or an equivalent combination of moderate- and vigorous-intensity activity. Aerobic activity should be performed in bouts of at least 10 min duration. For additional health benefits, adults should increase their moderate-intensity aerobic physical activity to 300 min per week, or engage in 150 min of vigorous-intensity aerobic physical activity per week, or an equivalent combination of moderate- and vigorous-intensity activity. Muscle-strengthening activities should be done involving major muscle groups on 2 or more days a week” (World Health Organization [WHO], 2017). Pursuant to the above-mentioned recommendations, the improvement of cardiorespiratory and muscular fitness as well as bone health are expected after introducing the appropriate amount of exercise. The risk of non-communicable diseases and depression would also be reduced (World Health Organization [WHO], 2017). It must be remembered that these suggestions are for the average population. Due to the specific conditions, physical activity in case of dentists should be tailored individually. For instance, to avoid muscle fatigue associated with the working position, exercises that strengthen the stabilizing muscle are recommended (Akuthota and Nadler, 2004). To provide proper core stability, only 10% of muscle contraction is needed (McGill, 2002). In the lumbar spine, low-intensity isometry and harmony of the deep muscles should be implemented, preferably by core strengthening, i.e., dynamic stability or torso neuromuscular training (McGill, 2002).

The study group declared 22 different types of imaging tests, including computed tomography and magnetic resonance imaging (Table 3). 86.6% of the respondents did not report these types of diagnostics (Table 3). Although most participants declared good health, the observed data is worrying. Perhaps the secondary type of prevention should be considered in dentistry students (Booth et al., 2011). It could mean the implementation of disease detection and management before any symptoms emerge, or the prevention of disability progression (Booth et al., 2011). Monitoring and screening tools play a crucial role in these methods (Booth et al., 2011).

Regarding structural disorders, no osteophytes, syndesmophytes or narrowing of the intervertebral holes were reported (Table 4). These results may suggest the functional nature of the disorder and an early phase of its development. Cases of diagnosed cervical spine instability, hypomobility, whiplash injury, dehydration of intervertebral discs or lowering of vertebral bodies were incidental. Some of them could be explained by trauma or conditioning of the body type. In people with discopathy and loss of cervical lordosis, the third type of prevention should be applied (Booth et al., 2011).

64.28% of the students correctly defined head protraction (Table 5). 13.39% were not familiar with the given terminology. Only 2.68% of the respondents consider protraction as a physiological position (Table 5). A random assessment of the cervical spine loads testified to insufficient job-related health knowledge (Table 5). 26.79% of people declared using a laptop or tablet and reading a book whilst lying on their back, 6.24% used a combination of lying on their back and other positions (Table 6). It should be assumed that lying on the back with a pillow could be a risk factor for pain. The position on the back induces head protraction and cervical spine flexion The range of the symptoms depends on the degree of cervical inclination and coincides with the previous observations of Ariëns et al. (2001). In most cases, this is accompanied by shoulder girdle protraction and increased kyphosis in the thoracic segment. On the other hand, lying on the front facing forward—depending on the situation—can lead to excessive extension in cervical segments. Combined twisting of the trunk in lying on the side, apart from pain symptoms, may contribute to disorders of the functioning of the motor apparatus of the eye and unilateral overload of the musculoskeletal system (Treleaven, 2008).

64.29% of the respondents performed practical tasks with patients or phantoms in the sitting position (Table 7). 26.78% of the students preferred a mixed manner of working (Table 7). No subjects worked with dental assistance, which was conditioned by the possibilities of practical training, including the technical capabilities of most universities. Students’ statements did not seem alarming, but it is difficult to assess whether, apart from the choice alone, the respondents actually properly adopted the declared postures. An incorrect sitting position can cause upper and/or lower crossed syndrome. The upper crossed syndrome is characterized by imbalance between the muscles of the head, neck and shoulder, with increased activity of pectoralis major and minor, trapezius, levator scapulae muscle, deltoid muscle, erector spinae muscles and suboccipital muscles (Kuć and Żendzian-Piotrowska, 2020). In the lower crossed syndrome, increased tension is applied to the iliopsoas, musculus rectus femoris, gastrocnemius, soleus, tibialis posterior, adductors of the thigh, musculus biceps femoris, semitendinosus and semimembranosus muscles, musculus tensor fasciae latae, musculus piriformis, erector spinae within the lumbar and thoracic region and musculus quadratus lumborum. Typical symptoms of this syndrome include weakness of the abdominal muscles (musculus rectus abdominis, musculus transversus abdominis, musculus obliquus abdominis), all gluteus muscles, musculus vastus lateralis et medialis, tibialis anterior and musculus peroneus longus et brevis (Page, 2006). The lower crossed syndrome decreases the corset muscle tone and is typically accompanied by the deepening of lumbar lordosis and the highlighting of the abdominal wall. In both syndromes, therapeutic treatment includes correction of peripheral disorders, restoration of muscle balance as well as improvement in stability and endurance in coordinated motion patterns (Page and Frank, 2007).

82.14% of the respondents used a dental mirror and binocular vision when treating cavities located on the palatal surface of the upper incisors. This may indicate excellent manual skills as well as optimal eye-hand coordination (Table 7). 14.29% of the students preferred direct binocular vision. Most likely these people worked in incorrect positions that forced combined movements of the cervical spine with lateral flexion and rotation as well as extension in the higher and flexion in the lower segments. Perhaps this is the result of insufficient coordination of the eye-hand complex or accompanying vision defects. However, it should be emphasized that extreme positions of the cervical spine may contribute to an increased tension of the suboccipital muscles, which may eventually lead to dysfunction, including the occurrence of myofascial pain. In turn, the use of a dental mirror or its absence in conjunction with monocular vision may cause abnormalities of vision and other problems.

Musculoskeletal symptoms increase stress level and affect physical and psychological condition, thus lowering the quality of life and work (Cherniack et al., 2010). Eighty three percent of dentists experience severe stress in their work (Baran, 2005). Around 60% see their profession as much more stressful than other jobs (Moore and Brødsgaard, 2001). Varela-Centelles et al. (2005) observed that 54.3% of dentists suffered from emotional exhaustion. In 56.6% of cases depersonalization was noted. 6.9% of the dentists demonstrated the lack of personal achievements. With respect to gender, a higher level of depersonalization was reported by males (Brake et al., 2003; Goehring et al., 2005). Myers and Myers (2004) noted that about 60% of dentists felt nervous and depressed. They lived under pressure and had difficulty sleeping. 58.3% of the study participants suffered from headache (Myers and Myers, 2004). 48.2% of the respondents were clearly tired for no apparent reason (Varela-Centelles et al., 2005). In 32% of the examined dentists a high level of minor psychiatric symptoms was noted (Myers and Myers, 2004). Humphris et al. (2002) reported that the level of emotional exhaustion was definitely higher in dentistry adepts than general medicine students. These authors emphasized that the level of stress and psychological disability were associated with the system of dentistry students’ education as well as immediate professional workload during their studies (Humphris et al., 2002).

In the presented study, the multiple linear regression model emphasized the link between Neck Disability Index (NDI), gender, Graded Chronic Pain Scale (GCPS v. 2.0) and Perceived Stress Scale (PSS-10) (Table 8). Regression coefficients highlighted independent contributions of each factor to the global evaluation of NDI (Table 8). GCPS showed a stronger association with NDI than gender and PSS-10 (higher standardized coefficients—β and *p*-value) (Table 8). PSS-10 remained in the 2nd place.

This model indicated that the risk of neck disability was higher in subjects with increased levels of chronic pain expressed by changes of GCPS v. 2.0., a higher level of perceived stress demonstrated by PSS-10 and females. Jay et al. (2015) reported that increased high amount of stress and musculoskeletal pain led to decreased work ability. Similar observations were noted by Marklund et al. (2020). These authors stressed a strong link between multi-site pain, high stress levels and reduced work productivity (Marklund et al., 2020). Moreover, pain is considered as a strong predictor for persistent stress and the resulting inconveniences in the future (Marklund et al., 2020). Other authors emphasized the connection between high stress levels and chronic facial pain as well as neck and back pain (Linton, 2000; Fillingim et al., 2011; Nevalainen et al., 2017).

The second model of multiple linear regression showed the interplay between the Graded Chronic Pain Scale (GCPS v. 2.0), standing position and the applied physiotherapy. Physiotherapeutic treatment procedures showed a stronger association with GCPS than the standing position (higher standardized coefficients—β and *p-*value) (Table 9). It means that in the case of dentistry students who declared participation in physiotherapy, higher levels of chronic pain (GCPS v. 2.0) could be expected. Moreover, the standing position during dental procedures decreases the tendency for graded chronic pain. This seems to be in accordance with previously reported observations. Callaghan and McGill (2001) and Dantas and de Lima (2015) demonstrated that complaints within the lower back are more pronounced in the sitting than standing position in dentists. These phenomena may induce fatigue of the deep layer of the erector spinae muscles responsible for maintaining a static position. This is a probable cause of initial back pain and can also be connected with chronic pain (Movahed et al., 2011).

## Strengths and Limitations

The results of this study reflect the need for holistic monitoring and screening among dentistry students with respect to job-related health problems. It is already possible to estimate the potential cost and benefits of treating dental occupational hazards in adepts. In each case, evaluation of individual patterns of work ergonomics, necessity for physical activity and cervical physiotherapeutic treatment needs is desirable and practicable. Our observations highlighted the need for primary, secondary and tertiary prevention (Booth et al., 2011). Studying dentistry should be preceded by verification of the risk factors and work load.

Self-reported questionnaires can be directed to a large group of people upon low financial cost. Due to the randomly sampled collection, these methods are often used as a primary screening tool in pilot studies. Bearing in mind that dentistry students are much closer to the aspects of ergonomics, physical activity and work-related musculoskeletal disorders, the information they provide tends to be more reliable. Despite the fact that the protocol used in the study did not contain sensitive questions, it is impossible to exclude the risk of social desirability bias.

## Conclusion

A relatively low incidence of structural abnormalities within the cervical spine and a highly diversified distribution of functional disorders exist among dentistry students. 26.79% of them required periodic therapeutic rehabilitation and for 34.82% it was advisable to implement regular physical activity. Due to professional conditions, medical and dental students are exposed to early cervical spine disorders. The priority is to prevent and relieve symptoms of musculoskeletal disorders resulting from long-lasting static positions. The factor influencing an early onset of such dysfunctions is an increased stress. An average level of pro-health awareness may be the reason for elevated cost of rehabilitation among this group of patients. Preventive actions in the workplace, proper physical exercise program as well as education in dental ergonomics should be provided. Due to the small sample of the study size further research is needed on the subject.

## Data Availability Statement

The raw data supporting the conclusions of this article will be made available by the authors, without undue reservation. Datasets are available on request.

## Ethics Statement

The studies involving human participants were reviewed and approved by the Bioethical Commission of the Medical University of Bialystok, Poland No. R-I-002/513/2017. Written informed consent for participation was not required for this study in accordance with the national legislation and the institutional requirements.

## Author Contributions

JK developed and planned the study. MŻ-P contributed to the interpretation of the results and supervised the project. JK and MŻ-P participated in the writing of the manuscript. Both authors discussed the results and revised the manuscript, as well as read and approved the submitted version.

## Conflict of Interest

The authors declare that the research was conducted in the absence of any commercial or financial relationships that could be construed as a potential conflict of interest.
